# PGC-1α or FNDC5 Is Involved in Modulating the Effects of Aβ_1−42_ Oligomers on Suppressing the Expression of BDNF, a Beneficial Factor for Inhibiting Neuronal Apoptosis, Aβ Deposition and Cognitive Decline of APP/PS1 Tg Mice

**DOI:** 10.3389/fnagi.2017.00065

**Published:** 2017-03-21

**Authors:** De-Yu Xia, Xin Huang, Chong-Feng Bi, Lin-Ling Mao, Li-Jun Peng, Hai-Rong Qian

**Affiliations:** ^1^Department of Neurology, General Hospital of NavyBeijing, China; ^2^Department of Neurology, Beijing Luhe Hospital, Capital Medical UniversityBeijing, China

**Keywords:** brain derived neurotrophic factor, PGC-1α, FNDC5, β-amyloid protein, Alzheimer's disease

## Abstract

Alzheimer's disease (AD) is generally defined as the aberrant production of β-amyloid protein (Aβ) and hyperphosphorylated tau protein, which are deposited in β-amyloid plaques (APs) and neurofibrillary tangles (NFTs), respectively. Decreased levels of brain-derived neurotrophic factor (BDNF) have been detected in patients with AD compared to control subjects. However, the underlying molecular mechanisms driving the downregulation of the BDNF remain unknown. Therefore, we explored the mechanisms underlying the regulation of BDNF in the neurons of APP/PS1 transgenic (Tg) mice, an AD experimental model. Using the APP/PS1 Tg mice, we found that BDNF expression was markedly downregualted at the age of 3- and 9-month-old. After cerebroventricular injection (i.c.v) of Aβ_1−42_ oligomers into the mice, BDNF was also found to be decreased, which demonstrated the critical roles of the Aβ_1−42_ oligomers in regulating the expression of BDNF. In neuronal culture, peroxisome proliferators-activated receptor γ coactivator 1α (PGC-1α) and fibronectin type III domain-containing 5 (FNDC5) were found to be downregulated by treatment with the Aβ_1−42_ oligomers. In addition, overexpression of either PGC-1α or FNDC5 reversed the suppressive effects of the Aβ_1−42_ oligomers on the expression of BDNF in neuroblastoma 2a (n2a) cells. More importantly, elevating the levels of PGC-1α, FNDC5 or BDNF in the n2a cells counteracted the effects of the Aβ_1−42_ oligomers on neuronal apoptosis. Additionally, intranasal administration BDNF in the APP/PS1 Tg mice decreased the Aβ deposition and reduced the cognitive decline of the mice.

## Introduction

Alzheimer's disease (AD) is characterized by the pathological production of β-amyloid protein (Aβ) and hyperphosphorylation of tau in β-amyloid plaques (APs) or neurofibrillary tangles (NFTs) (Bloom, 2014). Since Aβ accumulation multiple affects multiple biological functions, such as oxidative stress, neuroinflammation, neurotoxicity, autophagy and apoptosis (Yankner, 1996), the components of APs received a great amount of attention in prior works. For example, APs are essentially composed of Aβ_1−42_ (Glenner and Wong, 1984) and Aβ_23−35_ (Kubo et al., 2002; Gruden et al., 2007). As a consequence, different lengths of Aβ fragments greatly impair the normal physiological functions of the brain.

Brain derived neurotrophic factor (BDNF) has been identified as a versatile and multifunctional growth factor implicated in the control of a wide spectrum of adaptive processes in both the developing and adult brain, which range from the modulation of synaptic connectivity and excitation in neurodegeneration (Park and Poo, 2013). In patients with AD, the precursor form of BDNF and mature BDNF or its mRNA are decreased in the parietal cortex and hippocampus in the early stage of the disease (Phillips et al., 1991; Holsinger et al., 2000; Michalski and Fahnestock, 2003; Peng et al., 2005). In addition, serum levels of BDNF are also correlated with the severity of dementia (Laske et al., 2007). Interestingly, individuals with the Val66Met mutation in BDNF displays a decreased volume of specific brain regions and impaired learning ability (Egan et al., 2003). Blocking BDNF signaling with anti-TrkB antibodies induces cognitive decline in exercise animal models (Vaynman et al., 2006). For these reasons, BDNF has been suggested to control the aggregation of tau protein in NFTs (Murer et al., 1999). However, the roles of BDNF in Aβ production and deposition remain to be determined, especially the underlying mechanisms.

Recently, peroxisome proliferator-activated receptor γ coactivator 1α (PGC-1α) and fibronectin type III domain containing 5 (FNDC5) were previously shown to be responsible for regulating the expression of BDNF in mice (Wrann et al., 2013). PGC-1α was initially identified as a beneficial factor that was induced in skeletal muscle by exercise (Finck and Kelly, 2006). A subsequent study revealed the important role of PGC-1α in the inhibition of the neurodegeneration (Ma et al., 2010). PGC-1α has been suggested to exert neuroprotective effects against MPTP-induced Parkinson's disease (St-Pierre et al., 2006). In addition, PGC-1α also negatively regulates N-methyl-D-aspartate (NMDA) receptor activities, which results in reduction of excessive excitotoxicity in rat cortical neurons (Cheng et al., 2012). FNDC5 was identified as a PGC-1α-dependent myokine (Bostrom et al., 2012), that is expressed in the brain (Dun et al., 2013). In addition, a previous study showed that FNDC5 is located downstream of PGC-1α to modulate BDNF regulation in mice (Wrann et al., 2013).

Although these investigations have provided fragments of information about the potential connections between FNDC5/PGC-1α and BDNF, their potential contributions to AD still remain unknown. Therefore, we aimed to reveal the mechanisms of BDNF downregulation during the course of AD development and progression. In addition, the effects of BDNF on neuronal apoptosis, Aβ deposition and learning ability were further addressed.

## Materials and methods

### Reagents

Aβ_1−42_ was synthesized by Qiangyao Biotechnology (Shanghai, China). BDNF was obtained from Sigma-Aldrich Corp (St. Louis, MO, USA). Antibodies specific for BDNF, PGC-1α, and FNDC5 were obtained from Abcam (Shanghai, China). Antibodies against Aβ (Stock #2454) and β-actin were purchased from Cell Signaling Technology, Inc. (Danvers, MA, USA). Of note, the Aβ antibody specifically reacts with human Aβ. PGC-1α or FNDC5 cDNA plasmids were obtained from Origene Technologies (Rockville, MD, USA) and subcloned into the pCMV6-XL vector. All reagents for the qRT-PCR and SDS-PAGE experiments were purchased from Bio-Rad Laboratories. All other reagents are from Invitrogen (Carlsbad, CA, USA) unless otherwise specified.

### Cell culture

Mouse neuro-2a (n2a) cells were grown (37°C and 5% CO_2_) on 6-cm tissue culture dishes (10^6^ cells per dish) in DMEM supplemented with 10% FBS medium. To enhance the efficacy of transfection (Son et al., 2000), the cells were transfected with cDNA constructs of PGC-1α and FNDC5 in serum-free medium using lipofectamine 2,000, and the cells were maintained in serum-free medium for an additional 12 h before incubation with the Aβ_1−42_ oligomers.

### Transgenic mice and treatments

The wild-type (WT) and APP/PS1 [B6C3-Tg (APPswe, PSEN1dE9) 85Dbo/J (Stock Number: 004462)] Tg mice were obtained from The Jackson laboratory (Bar Harbor, ME, USA). Genotyping was performed at 3–4 weeks after birth. The mice were housed in a controlled environment under a standard room temperature, relative humidity and 12-h light/dark cycle with free access to food and water. The general health and body weight of the animals were monitored every day. The mouse brains from the different groups were collected under anesthesia and perfusion.

### Aβ_1−42_ oligomers preparation

The methods for preparing Aβ_1−42_ oligomers has been described previously (Pan et al., 2011). In brief, freeze-drying Aβ_1−42_ protein was initially monomerized by dissolution it to a final concentration of 1 μg/μl in 100% hexafluoroisopropanal (HFIP) and the solution was aliquoted in sterile eppendorf tubes. HFIP was then evaporated under vacuum and the peptide was stored at −20°C before reconstituent. For preparing Aβ_1−42_ oligomers, the peptide was initially resuspended in dimethylsulfoxide (DMSO) to 20 μg/μl with water bath ultrasonication for 10 min and the solution was then diluted to a final concentration of 0.2 μg/μl in phenol red-free F-12 media, and incubated at 4°C for 24 h.

### Real-time PCR

Real-Time PCR assays were performed with the MiniOpticon Real-Time PCR detection system (Bio-Rad) using total RNA and the GoTaq 1-step Real-Time PCR kit with SYBR Green (Promega) and the appropriate primers. The reaction mixtures were incubated at 50°C for 15 min and then 97°C for 5 min. Then, 35 PCR cycles were performed with the following temperature profiles: 97°C for 15 s, 58°C for 30 s, 68°C for 1 min, and 77°C for 1 min. Data were collected at the final step (77°C for 1 min) to prevent the inclusion of any fluorescence from primer dimers. The GenBank accession number and forward and reverse primers are as follows: mouse BDNF (NM_007540) F-TGAGCAAAGCCGAACTTCTC, R-TCACCTGGTGGAACATTGTG; PGC-1α (NM_008904) F-TGATGTGAATGACTTGGATACAGACA, R-GCTCATTGTTGTACTGGTTGGATATG; FNDC5 (NM_027402) F-ATGAAGGAGATGGGGAGGAA, R-GCGGCAGAAGAGAGCTATAACA, and GAPDH (NM_001289726) F-ACTCCACTCACGGCAAATTC, R-GGAGATGATGACCCTTTTGG. The gene expression values were normalized to those of GAPDH. Of note, the control group was always set as 1 and the experimental groups were compared to the control groups as previously described (Roberts et al., 2011).

### Western blots

The tissues or cells were lysed in radio-immune precipitation assay buffer (25 mM Tris-HCl [pH 7.6], 150 mM NaCl, 1% NP-40, 1% sodium deoxycholate, and 0.1% SDS) containing a protease inhibitor cocktail (Pierce Chemical Company, Shanghai, China). The protein content of the cell lysates was determined using a bicinchoninic acid (BCA) protein assay reagent (Pierce Chemical Company, Shanghai, China). The total cell lysates (4 μg) were subjected to SDS-PAGE, transferred to a membrane, and probed with a panel of specific antibodies. Each membrane was only probed with one antibody. β-actin was used as a loading control. All western blots were performed at least in triplicate using a different cell preparation each time.

### Intracerebroventricular injection

Aβ or vehicle (PBS) were intracerebroventricularly injected (i.c.v) into C57BL/6 mice. Briefly, stereotaxic injections were conducted at the following coordinates from the bregma: mediolateral: −1.0 mm; anteroposterior: −0.22 mm; and dorsoventral: −2.8 mm. Following the injections, each mouse recovered spontaneously on a heated pad. The reliability of the injection sites was validated by injecting trypan blue dye (Invitrogen) into separate cohorts of mice and observing staining in the cerebral ventricles. 24 h after the injection, the mouse brains were harvested after anesthesia and perfusion.

### Immunohistochemistry

Mouse brains were collected from the WT or APP/PS1 Tg mice and fixed with 4% paraformaldehyde. Serial sections (10 μM thick) were cut using a cryostat (Leica, CM1850, Germany). The slides were first rehydrated in a graded series of ethanol and submerged in 3% hydrogen peroxide to eliminate endogenous peroxidase activity. The levels of BDNF and Aβ were determined using an immunohistochemical staining kit with antibodies specific for BDNF (1:200 dilution in PB solution) and Aβ (1:200 dilution in PB solution), following the manufacturer's instructions (Invitrogen).

### Viability assay

Neuronal viability was determined by an MTT assay with minor modifications. In brief, n2a cells were incubated with an Ab in the absence or presence of BDNF. After the treatments, Tiler 96® Aqueous One Solution (20 μl) was added to the corresponding wells and the cells were further incubated for 4 h. The optical density was measured by a microplate reader (Bio-Rad, Bio-Rad Laboratories, Inc., Hercules, California, USA) at a wavelength of 490 nm.

### Flow cytometry

Annexin V-FITC/PI double staining was used to detect the apoptosis of n2a and SH-SY5Y cells. In detail, the cells were collected with an enzyme-free cell dissociation buffer and washed twice with cold PBS (−) before centrifugation at 2,500 rpm for 5 min (Thermo Fisher Scientific Inc., Waltham, MA, USA). The cell pellets were then resuspended in 500 μl binding solution with 5 μl annexin-V-fluorescein isothiocyanate and 5 μl of propidium iodide before the analysis with a FACS Calibur flow cytometer (Becton, Dickinson and Company, Franklin Lakes, New Jersey, USA).

### Morris water maze

The mice were trained and tested in a Morris water maze. In brief, the mice were pretrained in a circular water maze with a visible platform for 2 days. The platform was then submerged inside the maze, with the deck 0.5 cm below the surface of the water for the following experiments. Milk was added to the water to hide the platform from sight. The mice were placed inside the maze to swim freely until they found the hidden platform. The whole experiment took place over 7 days. For the first 6 days, the mice were placed in the maze with a maximum of 60 s to find the platform. The learning sessions were repeated with 4 trials each day, and an interval of 1 h between each session. The spatial learning scores (the latency period necessary to find and climb onto the hidden platform and the length of the path to the platform) were recorded. On the last day, the platform was removed, and the times that the mice passed through the memorized region were recorded for a period of 2 min (120 s). Finally, the recorded data were analyzed with a statistical program (ZH0065; Zhenghua Bioequipment, Yuanyang City, Henan, China).

### Animal committee

All animals were handled according to the guidelines of the Care and Use of Medical Laboratory Animals (Ministry of Health, Peoples Republic of China, 1994), and all experimental protocols were approved by the Laboratory Ethics Committees of the Navy General Hospital, China.

### Statistical analysis

All data are presented as the means ± S.E. of at least three independent experiments. The statistical significance of the differences between the means was determined with Student's *t*-test or one-way ANOVA, as appropriate.

## Results

### BDNF is downregulated in APP/PS1 Tg mice, and Aβ_1−42_ oligomers can decrease the expression of BDNF in the C57BL/6 mice

**Figure 1:**
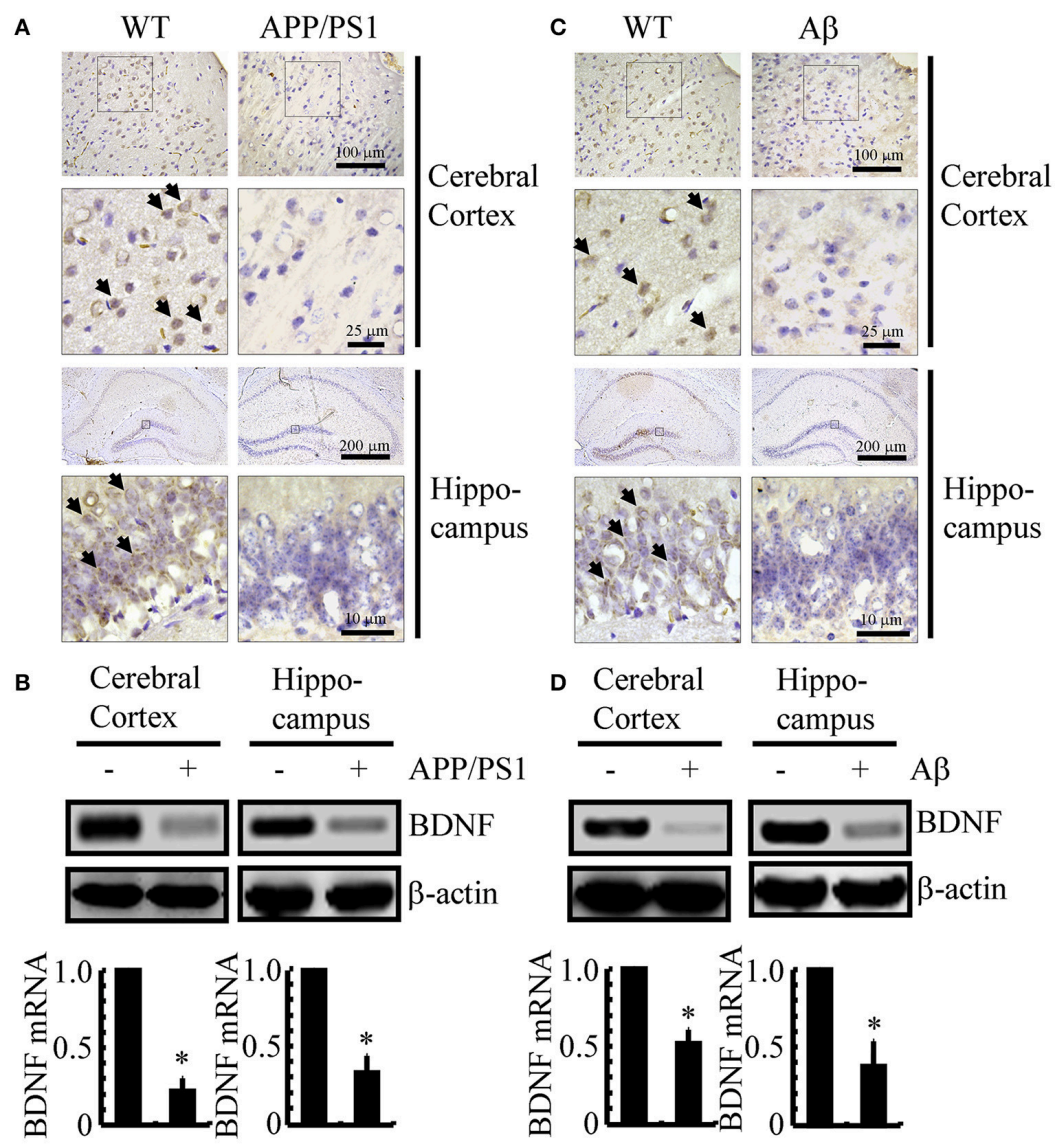
**The expression of BDNF was downregulated in APP/PS1 Tg mice and Aβ_1−42_ oligomer injection decreased the expression of BDNF in C57BL/6 mice. (A,B)** The brains of the 3-month-old APP/PS1 Tg mice were collected after anesthesia and perfusion (*n* = 8). **(C,D)** In select experiments, the Aβ_1−42_ oligomers (1 ng/5 μl) or vehicle (PBS) was injected (i.c.v) into the ventricles of the 3-month-old C57BL/6 mice (*n* = 8). **(A,C)** The immunoreactivity of BDNF was determined by IHC with a BDNF-specific antibody before the analysis with microscopy. **(B,D)** The mRNA and protein levels of BDNF were determined by qRT-PCR and western blots, respectively. GAPDH and β-actin served as the internal controls. The data represent the means ± S. E. of all the experiments. ^*^p < 0.05 compared with WT or vehicle-treated controls.

Since previous studies have shown that BDNF is associated with neuroprotective effects (Kitiyanant et al., 2012; Wang et al., 2015), we first determined the expression levels of BDNF in APP/PS1 Tg mice at 3 months of age. As shown in Figure 1A, BDNF immunostaining was evident in the cerebral cortex and hippocampus of 3-month-old C57BL/6 mice, and the positive staining was reduced in 3-month-old APP/PS1 Tg mice. To further confirm this finding, we examined the mRNA and protein levels of BDNF in the APP/PS1 Tg mice. In agreement with the immunostaining data, the mRNA and protein levels of BDNF were downregulated in both the cerebral cortex and hippocampus of the mice (Figure 1B and Supplemental Figure 1A). To reveal a possible reason for the downregulation of BDNF, we injected Aβ_1−42_ oligomers (i.c.v) into 3-month-old C57BL/6 mice. The results demonstrated that the Aβ_1−42_ oligomer injection (i.c.v) significantly suppressed the expression of BDNF in 3-month-old C57BL/6 mice (Figure 1C). Similar to these immunostaining results, the western blot and real-time PCR results also revealed the inhibitory effects of the Aβ_1−42_ oligomers on the expression of BDNF (Figure 1D and Supplemental Figure 1B). Consistent with these observations, our data revealed that BDNF expression is downregulated in APP/PS1 Tg mice, and BDNF expression is markedly decreased in C57BL/6J mice injected with exogenous Aβ_1−42_ oligomers, which supports a critical roles of Aβ_1−42_ oligomers in suppressing the expression of BDNF during the course of AD development and progression.

### Aβ_1−42_ oligomers decreased the expression of BDNF through a PGC-1α- or FNDC5-dependent mechanisms

**Figure 2:**
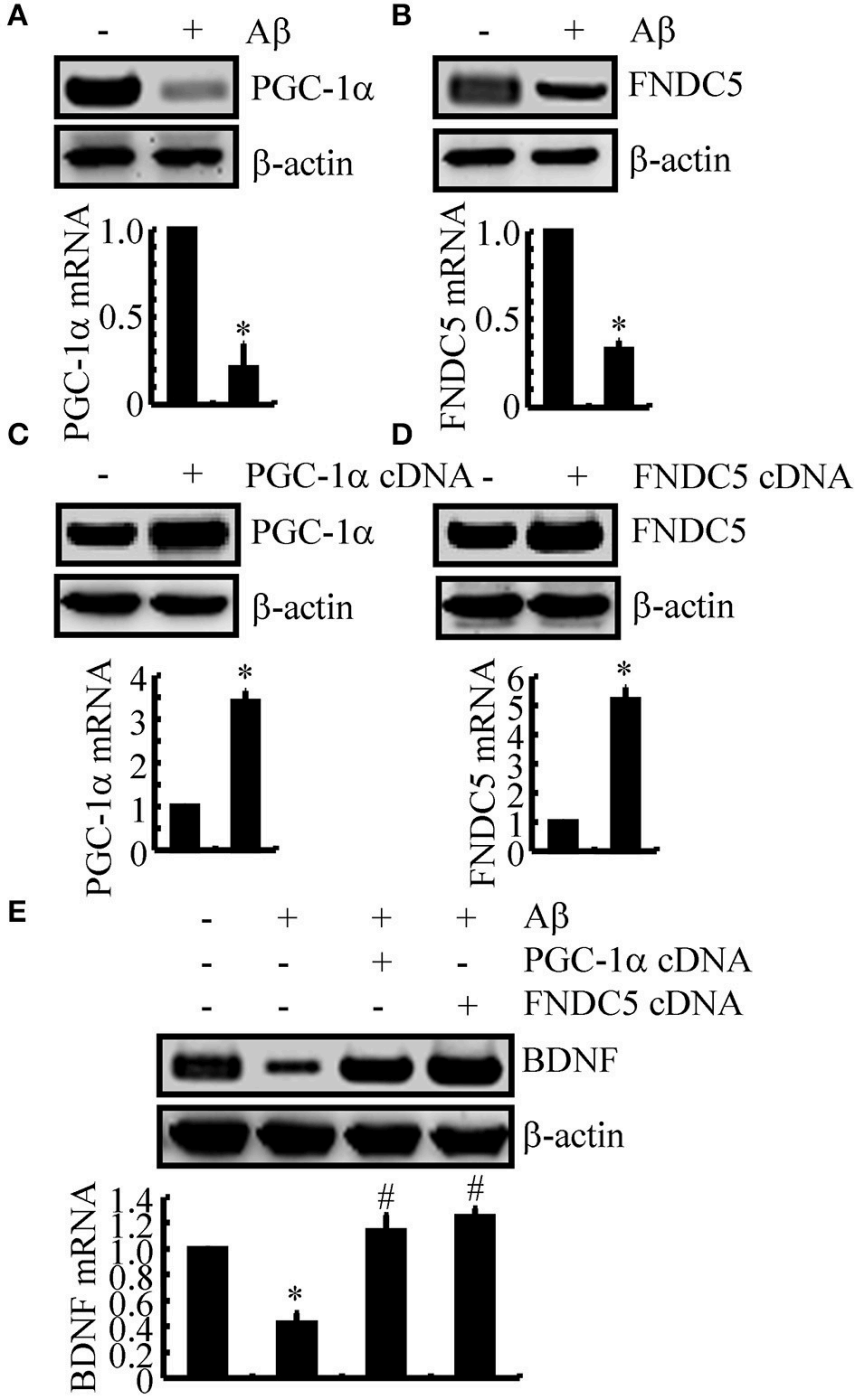
**The Aβ_1−42_ oligomers suppressed the expression of BDNF through a PGC-1α- and FNDC5-dependent mechanism**. **(A,B)** The n2a cells were treated with Aβ oligomers (1 ng/ml) for 48 h. **(C,D)** In select experiments, the n2a cells were transfected with either PGC-1α or FNDC5 cDNA for 48 h. **(E)** In separate experiments, the n2a cells were transfected with either PGC-1α or FNDC5 cDNA before the treatment with Aβ oligomers for 48 h. The mRNA and protein levels of BDNF, PGC-1α and FNDC5 were determined by qRT-PCR and western blots, respectively. GAPDH and β-actin served as the internal controls. The data represent the means ± S.E. of three times experiments. ^*^p < 0.05 compared with vehicle-treated or vector-transfected controls. ^#^*p* < 0.05 compared with Aβ-treated alone.

We next sought to elucidate the mechanisms of BDNF regulation in the APP/PS1 Tg mice. Since a previous study suggested that PGC-1α or FNDC5 are responsible for BDNF regulation (Wrann et al., 2013), we first evaluated the effects of Aβ oligomers on the expression of PGC-1α and FNDC5. To this end, n2a cells were treated with Aβ oligomers for 24 h. Treatment of the n2a cells with Aβ oligomers decreased the expression of PGC-1α and FNDC5 in the n2a cells (Figures 2A,B and Supplemental Figure 1C). To further elucidate the potential roles of PGC-1α and FNDC5 in regulating the expression of BDNF, we transfected the n2a cells with cDNA constructs of PGC-1α and FNDC5. The efficacy of transfection was confirmed by western blots and real-time PCR. The results demonstrated that PGC-1α and FNDC5 cDNA transfection significantly increased the mRNA and protein expression of corresponding genes (Figures 2C,D and Supplemental Figure 1D). Overexpression of the PGC-1α and FNDC5 markedly reversed the inhibitory effects of Aβ_1−42_ oligomers on the mRNA and protein expression of BDNF in the n2a cells (Figure 2E and Supplemental Figure 1E). Based on these findings, our findings demonstrated that Aβ_1−42_ suppressed the expression of BDNF through a PGC-1α- and FNDC5-dependent mechanism.

### Elevating the levels of PGC-1α, FNDC5 and BDNF alleviates the apoptotic effects of Aβ_1−42_ oligomers on neurons

**Figure 3:**
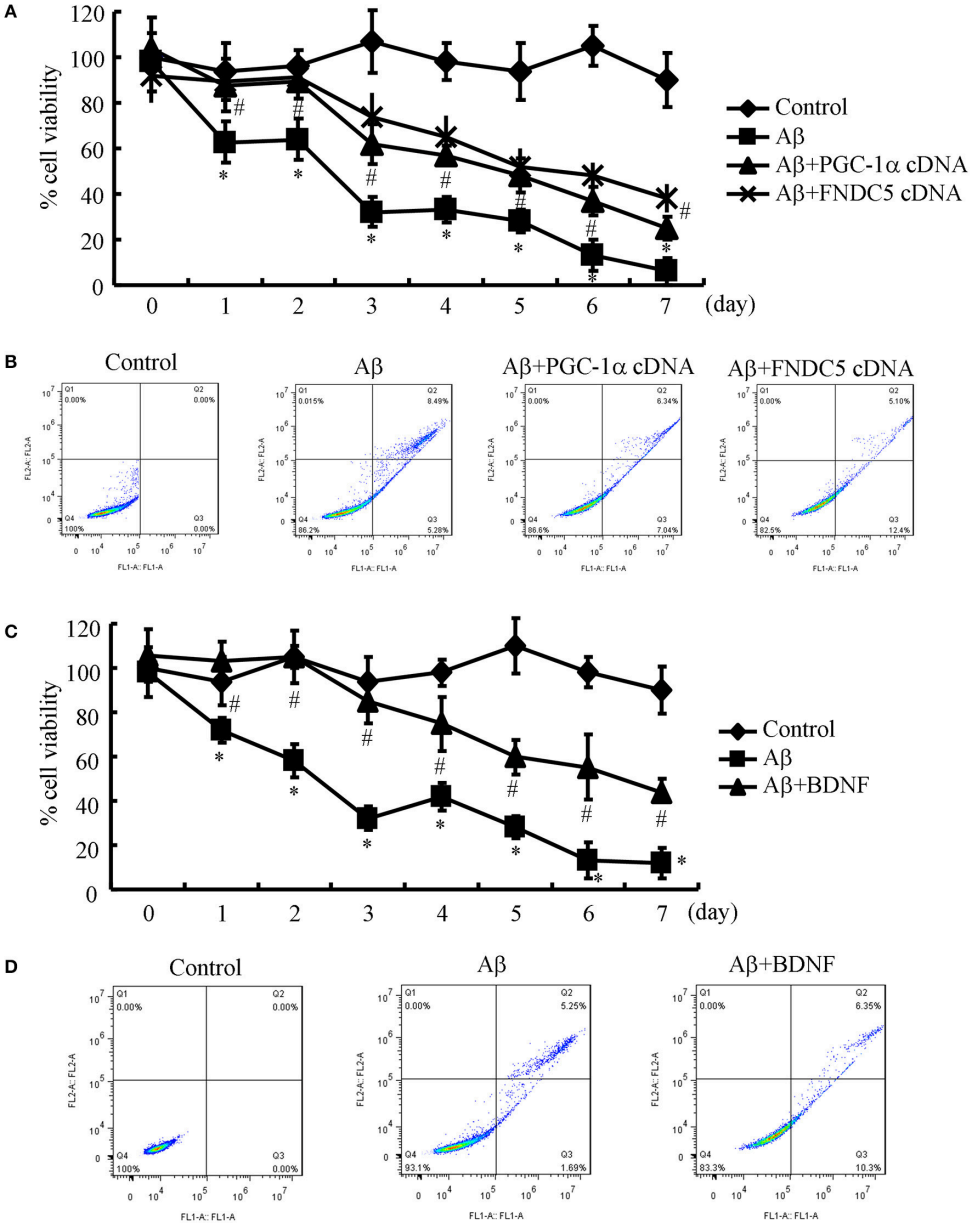
**Elevating the levels of PGC-1α, FNDC5 and BDNF partially protected neurons from Aβ_1−42_ oligomer-induced apoptosis. (A,B)** The neurons were incubated with Aβ (1 ng/ml) in the absence or presence of transfection with either PGC-1α or FNDC5 cDNA. **(C,D)** In select experiments, the n2a cells were incubated with Aβ (1 ng/ml) in the absence or presence of BDNF (1 ng/ml). **(A,C)** The viability of neurons in different groups was determined by an MTT assay. **(B,D)** The apoptosis of neurons was determined by flow cytometry after staining with PI and annexin V. The data represent the means ± S. E. of three times experiments. ^*^p < 0.05 compared with vehicle-treated or vector-transfected controls. ^#^*p* < 0.05 compared with Aβ-treated alone.

Since the Aβ_1−42_ oligomers are critical for suppressing the expression of BDNF in a PGC-1α- and FNDC5-dependent manner, we were prompted to elucidate the biological roles of BDNF in neurons. Therefore, the n2a cells were transfected with cDNA constructs of PGC-1α and FNDC5 before the treatment with the Aβ_1−42_ oligomers. Using an MTT assay, we found that the Aβ oligomers clearly suppressed neuronal viability (Figure 3A). More interestingly, the PGC-1α and FNDC5 overexpression significantly reduced the negative effects of the Aβ_1−42_ oligomers on neuronal viability (Figure 3A). To determine the mechanism of the observed neuronal death, the n2a cells were double stained with PI and annexin V after the indicated treatment. With flow cytometry, we found that the Aβ_1−42_ oligomers clearly induced neuronal apoptosis, which was reversed by the transfection with the PGC-1α and FNDC5 cDNA constructs (Figure 3B).

Due to the ability of PGC-1α and FNDC5 to modulate the expression of BDNF, we further determined the roles of BDNF in neuronal survival. To this end, we treated the n2a cells with the Aβ_1−42_ oligomers in the absence or presence of BDNF for the indicated times. The results demonstrated that BDNF treatment partially reversed the effects of the Aβ_1−42_ oligomers on neuronal death (Figure 3C). Similarly, BDNF also attenuated the apoptosis of the Aβ_1−42_ oligomer-stimulated n2a cells (Figure 3D). These observations clearly demonstrated that elevating the levels of PGC-1α, FNDC5 and BDNF ameliorates the effects of the Aβ_1−42_ oligomers on neuronal apoptosis.

### BDNF treatment suppresses the deposition of Aβ and reduces the cognitive decline of the APP/PS1 Tg mice

**Figure 4:**
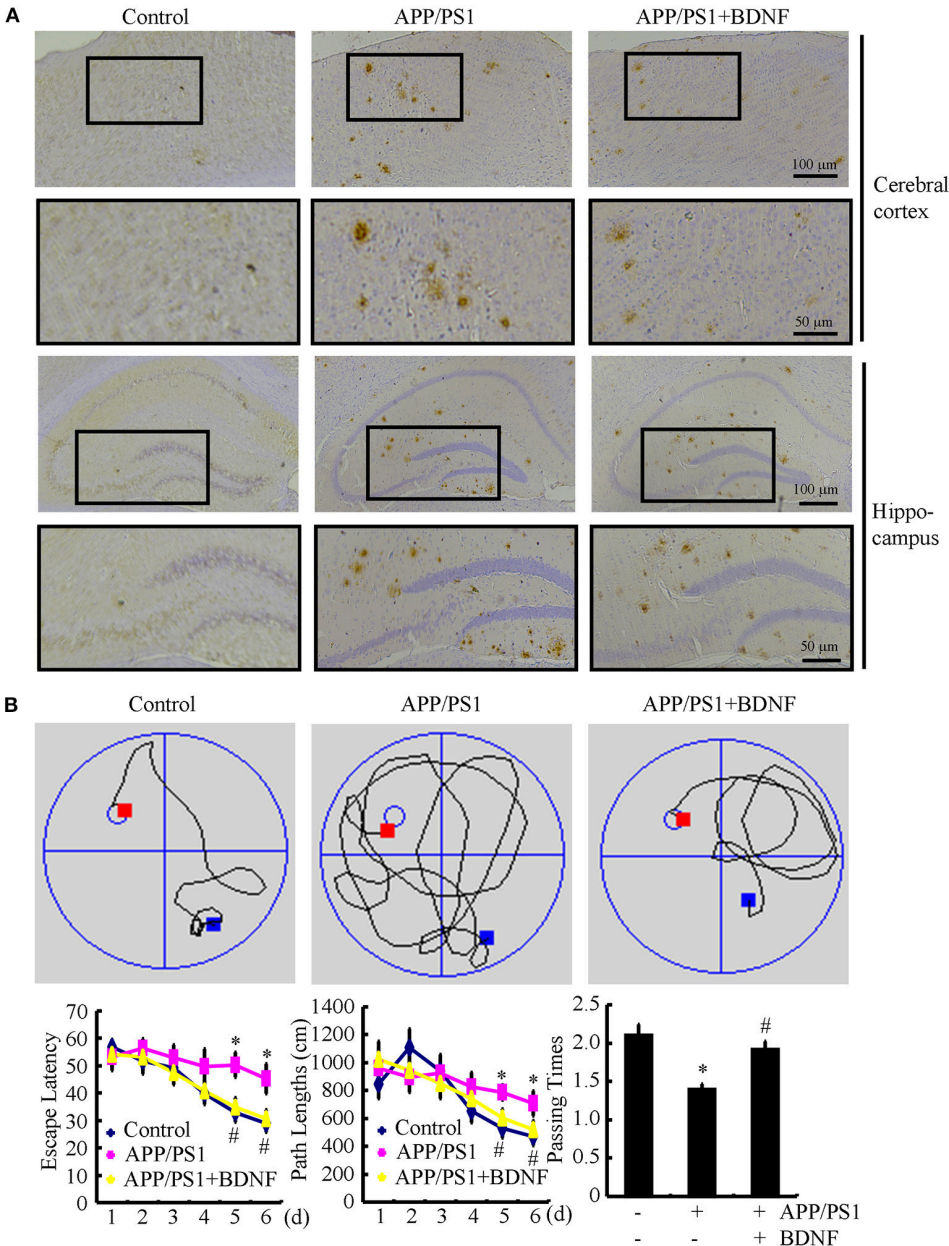
**BDNF treatment inhibits the deposition of Aβ and alleviates the cognitive decline of the APP/PS1 Tg mice**. The APP/PS1 Tg mice at 3 months of age received BDNF (1 ng/5 μl/d) intranasally for 6 months (*n* = 7). **(A)** The number of APs was determined by IHC using an Aβ-specific antibody. **(B)** The learning ability of the different groups of mice was determined by the Morris maze test. ^*^p < 0.05 compared with vehicle-treated controls. ^#^*p* < 0.05 compared with Aβ-treated alone.

Since BDNF counteracted the effects of the Aβ_1−42_ oligomers on neuronal apoptosis, we next sought to determine the roles of BDNF in the aggregation of Aβ and the cognitive decline of the APP/PS1 Tg mice. The results demonstrated that the deposition and aggregation of Aβ was highly induced in the APP/PS1 Tg mice and Aβ was deposited in the APs in the 9-month-old APP/PS1 Tg mice (Figure 4A). As BDNF is downregulated in 9-month-old APP/PS1 Tg mice (Supplemental Figure 2), we added back BDNF to the mice in order to determine the roles of BDNF in the deposition of Aβ. Of note, the BDNF treatment partially decreases the number of APs in the 9-month-old APP/PS1 Tg mice (Figure 4A). These observations demonstrate BDNF is beneficial for decreasing the deposition of Aβ in the APP/PS1 Tg mice.

Since we observed that the BDNF decreases the number of APs, we next investigated the relationship between brain BDNF levels and memory deficits in the APP/PS1 Tg mice. To this end, we assessed both spatial learning and memory abilities with the Morris water maze task. The results demonstrated that the untreated APP/PS1 Tg mice exhibited unequivocal learning deficits in the Morris water maze at 9 months of age (Figure 4B). The BDNF treatment improved the cognitive decline of the APP/PS1 Tg mice (Figure 4B). When we performed a probe test at 24 h after the last training trial, the untreated APP/PS1 Tg mice showed no preference for the target quadrant, which indicated significant memory impairment, whereas the BDNF-treated APP/PS1 Tg mice performed better than the non-treated APP/PS1 Tg mice (Figure 4B). These results clearly revealed that BDNF expression was downregulated in the APP/PS1 Tg mice and that the Aβ_1−42_ oligomers played critical roles in decreasing the expression of BDNF through a PGC-1α- and FNDC5-dependent mechanism. Reciprocally, elevating the levels of PGC-1α, FNDC5 and BDNF partially ameliorated the negative effects of the Aβ_1−42_ oligomers on neuronal survival. Additionally, the BDNF treatment clearly decreased the deposition of Aβ and reduced the cognitive decline of the APP/PS1 Tg mice.

## Discussion

**Figure 5:**
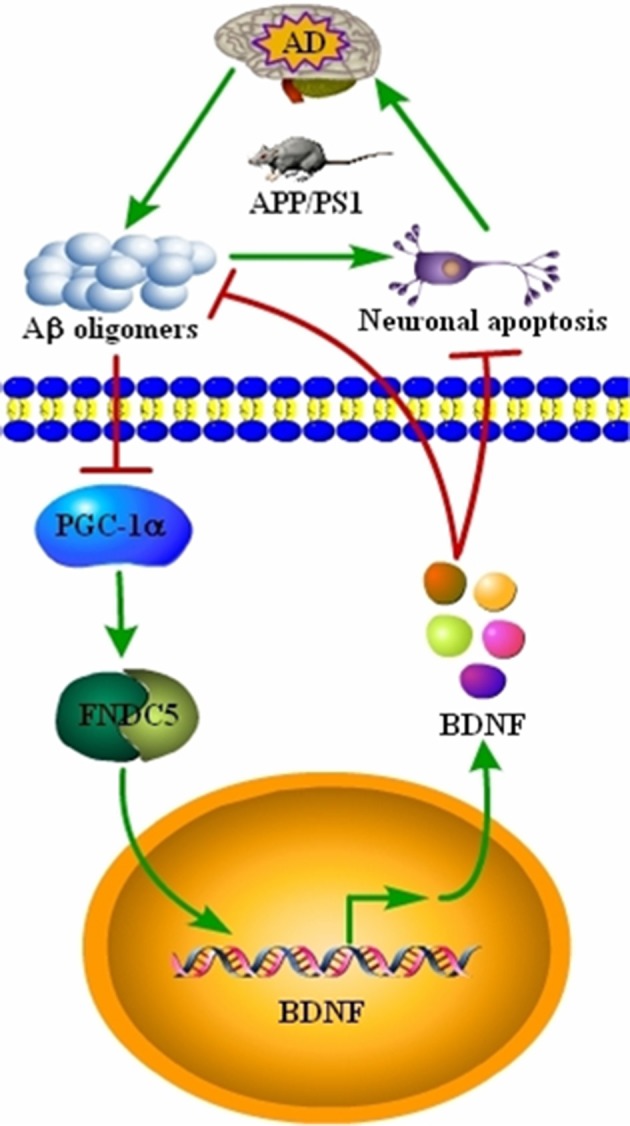
**Signaling events involved in BDNF regulation during the course of AD development and the critical roles of BDNF in inhibiting the apoptosis of neurons, Aβ deposition and cognitive decline of the APP/PS1 Tg mice**. BDNF expression was downregulated in the APP/PS1 Tg mice, and the Aβ_1−42_ oligomers are responsible for decreasing the expression of BDNF through a PGC-1α- and FNDC5-dependent mechanism. Reciprocally, elevating the levels of PGC-1α, FNDC5 and BDNF alleviated the effects of the Aβ_1−42_ oligomers on neuronal apoptosis. More importantly, BDNF treatment decreased the deposition of Aβ and reduced the cognitive decline of APP/PS1 Tg mice.

AD is characterized by the aberrant production of Aβ and hyperphosphorylated tau, which aggregate in APs and NFTs, respectively (Bloom, 2014). Recently, BDNF expression was found to be downregulated in conjunction with the development and progression of AD (Phillips et al., 1991; Holsinger et al., 2000; Michalski and Fahnestock, 2003; Peng et al., 2005). However, the underlying mechanisms of the relationship between BDNF and AD remained unknown. Therefore, we sought to determine the mechanisms of BDNF downregulation, and the beneficial effects of BDNF on AD. Indeed, the Aβ_1−42_ oligomers have the ability to inhibit the expression of BDNF through a PGC-1α- and FNDC5-dependent mechanism. Reciprocally, treatment of neurons with exogenous BDNF prevents the Aβ_1−42_ oligomers-induced neuronal apoptosis. Additionally, the BDNF treatment has the ability to alleviate the aggregation of Aβ and reduce the cognitive decline of the APP/PS1 Tg mice (Figure 5).

BDNF expression is tightly regulated in physiological conditions. In AD patients, BDNF expression was shown to be downregulated in conjunction with the development and progression of AD (Phillips et al., 1991; Holsinger et al., 2000; Michalski and Fahnestock, 2003; Peng et al., 2005). In agreement with these prior works, we found that the expression of BDNF was downregulated in the APP/PS1 Tg mice. More interestingly, we further identified the Aβ_1−42_ oligomers as the key factors that inhibit the expression of BDNF in the APP/PS1 Tg mice. Consistent with our observations, Lattanzio et al. (2016) reported that Aβ_25−35_ has ability to suppress the expression of BDNF in SH-SY5Y cells. Furthermore, Aβ oligomers are able to reduce the expression of BDNF in the APOE4 Tg mice (Sen et al., 2015). Thus, the aggregation of Aβ may be critical for downregulating the expression of BDNF during the course of AD development and progression.

In addition to our finding that the Aβ_1−42_ oligomers exert suppressive effects on the expression of BDNF, we found that PGC-1α and FNDC5 are involved in regulating the expression of BDNF. In agreement with our observation, PGC-1α and FNDC5 were demonstrated to be critical for the synthesis of BDNF in the brains of mice (Wrann et al., 2013). Although the current findings are quite limited, PGC-1α has been suggested to play an important role in the brain. For example, a lack of PGC-1α in the brain is associated with neurodegeneration (Lin et al., 2004; Ma et al., 2010). Outside of the context of AD, PGC-1α has also been reported to maintain neuronal dendritic spines (Cheng et al., 2012). Apart from PGC-1α, FNDC5 has been reported to trigger the differentiation of PC12 cells into neuronal cells (Ostadsharif et al., 2011). Knockdown of FNDC5 expression in neuronal precursors impairs their development into mature neurons, which suggests a beneficial role for FNDC5 in neurons (Hashemi et al., 2013). Therefore, we added to the previous body research and determined that the Aβ_1−42_ oligomers suppress the expression of BDNF through a PGC-1α- and FNDC5-dependent mechanism.

The Aβ_1−42_ oligomers are neurotoxic, so it is easier to demonstrate the neuroprotective effects of BDNF on neurons via disrupting the aggregation of APs. Indeed, BDNF exerted protective effects on neurons and blocked Aβ_1−42_-induced apoptosis. Consistent with our observations, Aβ exhibited dose-dependent toxicity in cortical neurons (Geci et al., 2007). In addition, BDNF has been reported to protect neurons from cellular damage (Lindvall et al., 1994). In animal models, BDNF was suggested to protect neurons from tau-induced impairment (Jiao et al., 2016). Furthermore, BDNF was reported to rescue neurogenesis in Aβ-injured neurons (Kitiyanant et al., 2012; Wang et al., 2015). These observations support our data showing that BDNF prevents neuronal death via inhibiting apoptosis in the Aβ_1−42_ oligomers-treated neurons.

Based on this finding, mutating BDNF or blocking its receptor, TrkB, were shown to clearly impair learning ability in the brains of animal models (Egan et al., 2003; Vaynman et al., 2006). The levels of BDNF in serum are also associated with the severity of dementia (Laske et al., 2007). In a model of AD, upregulation of BDNF ameliorates the cognitive decline of rats (Li et al., 2016). Consistent with this observation, a series of investigations have demonstrated that the cognitive decline of the APP/PS1 or 3XTg-AD mice was improved once the expression of BDNF was upregulated by different stimuli (Corona et al., 2012; Xiang et al., 2014). In our study, BDNF played pivotal roles in suppressing the deposition of Aβ and reducing cognitive decline of the APP/PS1 Tg mice.

## Author contributions

DX and XH conceived and performed all of the experiments, participated in the design of the study and wrote the manuscript. CB, LM, and LP carried out select experiments. HQ interpreted the data and wrote the manuscript.

### Conflict of interest statement

The authors declare that the research was conducted in the absence of any commercial or financial relationships that could be construed as a potential conflict of interest.
