# Supplementary material for: PGC-1α or FNDC5 Is Involved in Modulating the Effects of Aβ1−42 Oligomers on Suppressing the Expression of BDNF, a Beneficial Factor for Inhibiting Neuronal Apoptosis, Aβ Deposition and Cognitive Decline of APP/PS1 Tg Mice
Source: Front Aging Neurosci. 2017 Mar 21;9:65. doi: 10.3389/fnagi.2017.00065 (PMC5359257; doi:10.3389/fnagi.2017.00065)
Supplement: Supplementary file 1 [file Image1.PDF]

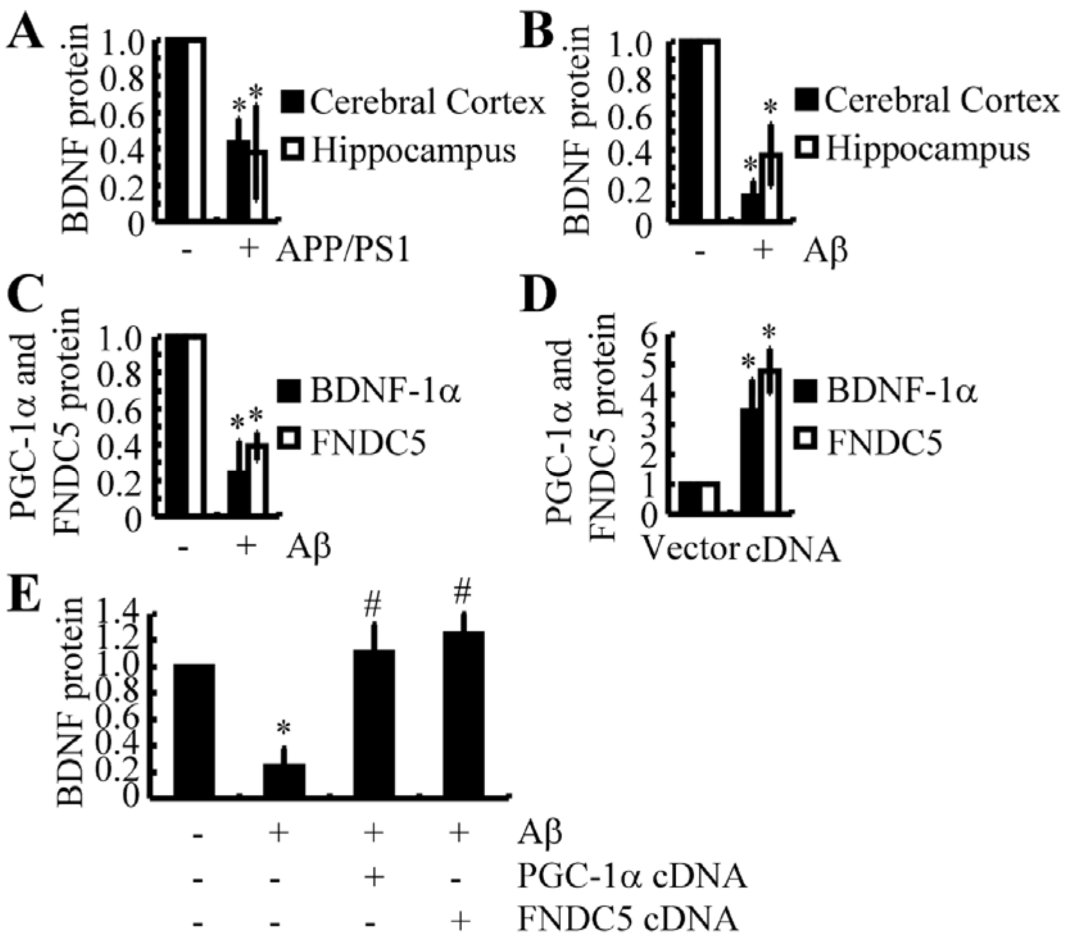

Supplemental Figure 1. Analysis of western blots. (A) The brains of 3-month-old APP/PS1 Tg mice were collected after anesthesia and perfusion. (B) A$\beta_{1\text{-}42}$ oligomers (1 ng/5 μl) or vehicle (PBS) was injected (i.c.v) to the ventricles of 3-month-old C57BL/6 mice. (C) n2a cells were treated with Aβ oligomers (1 ng/ml) for 48 h. (D) n2a cells were transfected with either PGC-1α or FNDC5 cDNA for 48 h. (E) n2a cells were transfected with either PGC-1α or FNDC5 cDNA before treating with Aβ oligomers for 48 h. The protein was extracted by RIPA buffer and the expression of corresponding protein was probed by western blots. The intensity of bands was quantified relative to β-actin for each treatment using the Bio-Rad gel image system. The data represent the means ± S.E. of all the experiments. *, *p<0.05* compared with WT, vehicle-treated or vector-transfected controls. #, *p<0.05* compared with Aβ-treated alone.
