# Supplementary material for: PGC-1α or FNDC5 Is Involved in Modulating the Effects of Aβ1−42 Oligomers on Suppressing the Expression of BDNF, a Beneficial Factor for Inhibiting Neuronal Apoptosis, Aβ Deposition and Cognitive Decline of APP/PS1 Tg Mice
Source: Front Aging Neurosci. 2017 Mar 21;9:65. doi: 10.3389/fnagi.2017.00065 (PMC5359257; doi:10.3389/fnagi.2017.00065)
Supplement: Supplementary file 2 [file Image2.PDF]

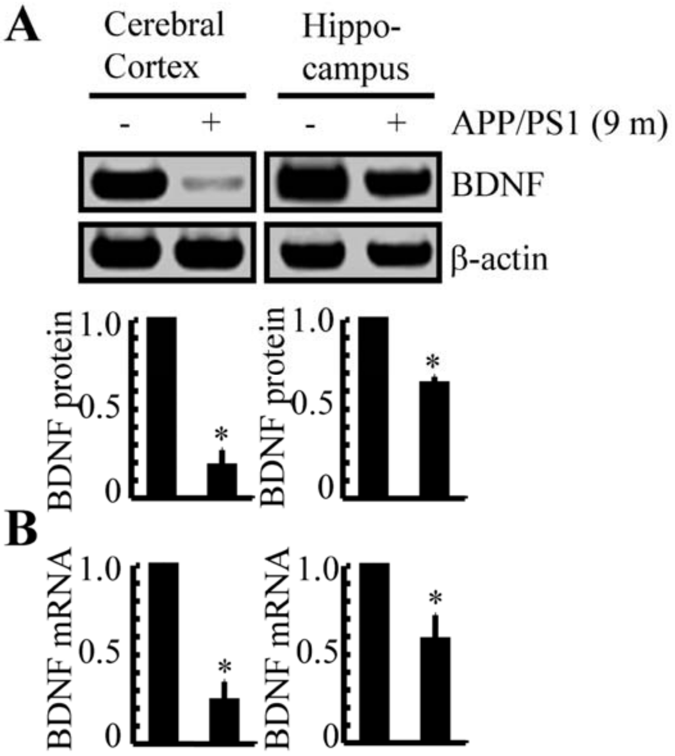

Supplemental Figure 2. The expression of BDNF was downregulated in 9-month-old APP/PS1 Tg mice. The brains of the 9-month-old APP/PS1 Tg mice were collected after anesthesia and perfusion. The mRNA and protein levels of BDNF were determined by qRT-PCR and western blots, respectively. GAPDH and β-actin served as the internal controls. The data represent the means ± S. E. of all the experiments. *$p<0.05$ compared with WT controls.
